# Relationship between socio-demographic correlates and human development index with physical activity and sedentary time in a cross-sectional multicenter study

**DOI:** 10.1186/s12889-022-13117-9

**Published:** 2022-04-06

**Authors:** Gerson Ferrari, Claudio Farías-Valenzuela, Juan Guzmán-Habinger, Clemens Drenowatz, Adilson Marques, Irina Kovalskys, Georgina Gómez, Attilio Rigotti, Lilia Yadira Cortés, Martha Cecilia Yépez García, Rossina G. Pareja, Marianella Herrera-Cuenca, Priscila Marconcin, Javiera Lobos Chávez, Mauro Fisberg

**Affiliations:** 1 Universidad de Santiago de Chile (USACH), Escuela de Ciencias de la Actividad Física, el Deporte y la Salud, Chile, Las Sophoras 175, Estación Central, Santiago, Chile; 2 Laboratorio de Rendimiento Humano, Grupo de Estudio en Educación, Actividad Física y Salud (GEEAFyS), Universidad Católica del Maule, Talca, Chile; 3 Instituto del Deporte, Universidad de las Américas, 9170022 Santiago, Chile; 4 Facultad de Ciencias, Universidad Mayor, Santiago, Chile; 5 Facultad de Ciencias, Especialidad medicina del deporte y la actividad física, Universidad Mayor, Santiago, Chile; 6 Division of Sport, Physical Activity and Health, University of Education Upper Austria, Linz, Austria; 7 CIPER, Faculdade de Motricidade Humana, Universidade de Lisboa, Lisbon, Portugal; 8 ISAMB, Faculdade de Medicina, Universidade de Lisboa, Lisbon, Portugal; 9 Carrera de Nutrición, Facultad de Ciencias Médicas, Pontificia Universidad Católica Argentina, Buenos Aires, Argentina; 10 Departamento de Bioquímica, Escuela de Medicina, Universidad de Costa Rica, San José, Costa Rica; 11 Centro de Nutrición Molecular y Enfermedades Crónicas, Departamento de Nutrición, Diabetes y Metabolismo, Escuela de Medicina, Pontificia Universidad Católica, Santiago, Chile; 12 Departamento de Nutrición y Bioquímica, Pontificia Universidad Javeriana, Bogotá, Colombia; 13 Colégio de Ciencias de la Salud, Universidad San Francisco de Quito, Quito, Ecuador; 14 Instituto de Investigación Nutricional, La Molina, Lima, Peru; 15 Centro de Estudios del Desarrollo, Universidad Central de Venezuela (CENDES-UCV)/Fundación Bengoa, Caracas, Venezuela; 16 Datrics, Santiago, Chile; 17 Centro de Excelencia em Nutrição e Dificuldades Alimentaes (CENDA), Instituto Pensi, Fundação José Luiz Egydio Setubal, Hospital Infantil Sabará, São Paulo, Brazil; 18 Departamento de Pediatria da Universidade Federal de São Paulo, São Paulo, Brazil

**Keywords:** Human development index, Sedentary time, Physical activity, International study

## Abstract

**Background:**

Socio-demographic correlates and human development index (HDI) are associated with self-reported physical activity, but only a few studies have focused on device-measured physical activity and sedentary time in Latin America. We examined the relationship between socio-demographic correlates and HDI with physical activity and sedentary time in a cross-sectional study.

**Methods:**

We based our analyses on 2522 (53.1% women; 18–65 years [mean age 38.3 years]) adults drawn from the eight Latin America countries. Physical activity (light, moderate, vigorous, and moderate-to-vigorous intensity and steps) and sedentary time were assessed using Actigraph GT3X + accelerometers. Sex, age, and race/ethnicity were self-reported. The HDI country information was obtained from the United Nations Development Program.

**Results:**

For the age, ethnicity, vigorous physical activity and steps/day, there were significant differences between high and very high HDI countries. Women and younger age presented lower sedentary time than men and older. In moderate-to-vigorous physical activity, we found lower duration in women (-13.4 min/week), younger age (-0.1 min/week), and white/caucasian (-2.7 min/week) than men, older ages and mixed ethnicity. Women (-1266.5 steps/week) and very high HDI (-847.3 steps/week) presented lower steps than men and high HDI. Black (2853.9 steps/week), other (1785.4 steps/week), and white/caucasian ethnicity (660.6 steps/week) showed higher steps than mixed ethnicity.

**Conclusions:**

Different socio-demographic correlates are associated with physical activity intensity; however, HDI is associated with vigorous physical activity and steps in the Latin American region, which can in turn guide policies to promote physical activity in the region.

**Trial registration:**

ClinicalTrials.GovNCT02226627. Retrospectively registered on August 27, 2014.

## Background

High levels of physical activity and limited sedentary behavior have been associated with numerous health benefits, affecting not only high-income nations but also low- and middle-income countries [1–3]. One of the problems that affect both middle- and high-income countries is physical inactivity [4].

Physical activity is a complex behavior regulated by both individual and contextual factors [5]. Within the contextual factors, it has been described that the levels of physical activity are influenced by socio-demographic variables [6, 7]. An indicator that allows comparing countries considering these key aspects is the Human Development Index (HDI). The United Nations Development Programme describes the HDI as "a measure of average achievement in key dimensions of human development: a long and healthy life, being knowledgeable and having a decent standard of living" [8]. The HDI is composed of education, estimated as the expected years of schooling and average years of schooling; income or standard of living, estimated as gross national income per capita; and estimated health based on life expectancy at birth in years [9]. An advantage of the HDI is that it allows analytical comparisons between countries [10]. Countries with a higher HDI have been described as having higher levels of physical inactivity [11]. In Europe, Cameron et al. [12] found that higher socio-demographic correlates were associated with leisure-time physical activity but not with objectively measured physical activity intensity. These findings indicate that socio-demographic factors and HDI can be associated with physical activity intensity.

When the relationship between HDI and physical activity has been studied, physical activity was self-reported [13, 14], which is at risk of reporting bias [15]. Additional information on the association between HDI and objectively determined physical activity is, therefore, warranted. Unfortunately, there are few accelerometer data measured in Latin America since they are more expensive than subjective self-report methods [7]. Furthermore, there are large differences between self-reported and device-measured physical activity and sedentary time values [15]. Accordingly, correlation coefficients between minutes of physical activity and sedentary time from accelerometry and subjective self-report methods are low [15, 16].

At the same time, less evidence is available regarding socio-demographic and HDI in Latin American countries. Therefore, in a cross-sectional multicenter study, the present study aimed to determine the relationship between socio-demographic correlates and HDI with physical activity and sedentary time.

## Methods

### Study design and sample

The data for the current study was captured from the Latin American Study of Nutrition and Health (*Estudio Latinoamericano de Nutrición y Salud*, ELANS), which was conducted from 2014 to 2015 using a common design and comparable methods across countries. ELANS is a cross-sectional, epidemiological, multi-national survey that uses a large representative sample (15 to 65 years old) from eight countries (i.e., Argentina, Brazil, Chile, Colombia, Costa Rica, Ecuador, Peru, and Venezuela) and focuses on urban populations. The ELANS protocol is registered in ClinicalTrials.gov (#NCT02226627) and was approved by the Western Institutional Review Board (#20,140,605). Ethical approval to conduct this study was obtained from the ethical boards at each study site. This research was performed according to the ethical principles from the Declaration of Helsinki. Informed consent/assent was obtained from all participants before data collection. Full details of the ELANS (https://www.elansstudy.com/), are available elsewhere [5, 17].

To obtain representative samples, a complex and multistage clustered sampling design method was used, representing all regions for each country and randomly selecting the main cities. In each country, stratified recruitment of sample was done across sex, age, and socioeconomic status. In total, 92 cities participated in the ELANS (seven to 23 cities in each country). Details about participant sampling and recruitment strategies have been published elsewhere [5, 17].

A total of 9218 (4409 men) participants were included in the ELANS study. The sample with accelerometer data included 2732 participants aged 15–65 years, representing 29.6% of the total ELANS participants (*N* = 9218). Adolescents between 15 to 17 years were excluded from the current analyses as ELANS did not include adolescents who were younger than 15 years and because this study focused on the adult population. Therefore, the current manuscript only examines adults between 18 and 65 years of age, resulting in a final sample of 2522 participants. Details have been published elsewhere [5, 17].

### Socio-demographics correlates

Sex (men and women), age (18 to 65 years), and race/ethnicity were collected for all participants using standard questionnaires during face-to-face interviews. Participants were asked about their race/ethnicity (mixed/caucasian, black, white, and other [i.e., Asian, Indigenous, Gypsy, and other]). Mixed/caucasian was defined as being born of a father and mother of different races/ethnicities. Further details can be found in a previous study [18].

### Country human development index

The HDI is a composite index, ranging from 0 to 1, calculated using education, life expectancy, and per capita income [19]. This index was created by the United Nations Development Programme to rank countries on a scale of human development conceptualized in terms of the capabilities of humans within the countries to function [20].

The HDI information was obtained from the United Nations Development Programme, and the classification of the country was used categorically according to the original classification (low, medium, high or very high) [19, 21]. The participant’s countries were classified as high(0.70 to 0.79) or very high (≥ 0.80) HDI.

### Physical activity and sedentary time

The Actigraph GT3X + accelerometers (Fort Walton Beach, FL, United States) were used to objectively monitor mean min/week of sedentary time, as well as the complete range of intensities of physical activity (including light, moderate, vigorous and moderate-to-vigorous physical activity), steps and sedentary time. The Actigraph GT3X + provides reliable and valid estimates of sedentary time, physical activity, and steps [22–24].

Data was collected via two home visits. In the first home visit, the designated participants received the accelerometer with instructions that contained a brief description of the device, details of how to wear it, and contact information. In addition, participants were given a diary to record wear-time per day for the following seven consecutive days. The accelerometer and diary were retrieved at the second home visit.

Participants were asked to wear the accelerometer while awake and remove them only for showering/bathing or other water activities and when sleeping. Participants were asked to wear the accelerometer on an elasticized belt at hip level on the right mid-axillary line. Participants were included in the analysis if they had at least five valid days of data with at least 10 h/day of wear time, including at least one weekend day. After excluding the nocturnal sleep period time, periods with more than 60 min of consecutive zero accelerometer counts were categorized as non-wear time [25]. The mean valid days of wear time and mean the number of hours of daily wear time within the analyzed sample with five or more valid days was 6.6 (95% confidence interval [CI]: 6.2; 7.0) and 15.3 h/day (95%CI: 15.1; 15.5), respectively [7].

The research team went to the participants’ homes to retrieve the devices following the 7-day measurement period. The team downloaded the data using the latest version of the ActiLife software (version 6.0; ActiGraph, Pensacola, FL). Data were collected at a sampling rate of 30 Hz and processed using 60-s epochs without the use of a filter [26]. Sedentary time was defined as time accumulated at < 100 activity counts/min, ≥ 101–1951 activity counts/min for light physical activity, ≥ 1952–5724 activity counts/min for moderate physical activity, ≥ 5725 activity counts/min for vigorous physical activity, and ≥ 1952 activity counts/min for moderate-to-vigorous physical activity [27, 28]. Additionally, we estimated the mean of steps count. Participants were categorized as meeting (≥ 150 min/week) or not meeting (< 150 min/week) MVPA guidelines as defined by the World Health Organization [29].

### Statistical analysis

The Kolmogorov–Smirnov test and histograms were used to check data normality distribution. Descriptive statistics included absolute and relative frequencies, medians and interquartile range (IQR: 25th and 75th). High and very high HDI countries were compared using Mann–Whitney (continuous variables) and chi-square test (categorical variables).

Linear regression models (β unstandardized coefficient and 95% confidence intervals: 95%CI) were used to examine the relationship between socio-demographic correlates characteristics (sex, age and ethnicity) and HDI. Analysis were mutually adjusted for each other with sedentary time, physical activity intensity (min/week; sedentary time, light, moderate, vigorous, and moderate-to-vigorous) and steps/week. We also adjusted all models for countries, regions, and cities. All analyses were performed using SPSS V27 software (SPSS Inc., IBM Corp., Armonk, New York, NY, USA). A significance level of 5% was adopted.

## Results

There were no significant differences between the participants who were asked to wear an accelerometer and those who did not. The descriptive characteristics of the participants (*n* = 2522; 53.1% women; 18–65 years [mean age 38.3 years]) are shown in Table 1, stratified by country. Overall, 51.2% of participants were classified as mixed/caucasian ethnicity. The median sedentary time, light, moderate, vigorous, moderate-to-vigorous physical activity (min/day), and steps (counts/day) were 561.0, 306.1, 27.7, 0.0, 28.3, and 9697.8, respectively. The prevalence of not meeting moderate-to-vigorous physical activity guidelines was 35.8%.

**Table 1:** Demographic characteristics and device-measured physical activity and sedentary time, in overall and by coutnry

Variables	Overall	Argentina	Brazil	Chile	Colombia	Costa Rica	Ecuador	Peru	Venezuela
**Sample size** (n)	2522	271	522	274	319	247	249	302	338
**Age** (years, mean (SD))	38.3 (13.4)	40.7 (13.0)	39.1 (13.3)	38.7 (13.2)	39.5 (13.9)	38.5 (12.8)	36.5 (13.6)	37.1 (13.4)	36.1 (13.2)
**Sex** (%)									
Men	46.9	42.1	44.1	46.4	49.8	47.4	50.2	47.0	49.7
Women	53.1	57.9	55.9	53.6	50.2	52.6	49.8	53.0	50.3
**Ethnicity (%)**									
Mixed/caucasian	51.2	28.3	19.1	70.0	58.3	37.2	91.6	89.5	44.8
Black	6.7	7.3	20.3	1.7	6.6	2.8	1.6	1.0	5.1
White	34.9	61.6	42.0	26.1	29.1	48.4	3.6	7.5	44.8
Other	7.2	2.8	18.6	2.2	6.0	11.6	3.2	2.0	5.3
**Device-measured (median [IQR])**									
Sedentary time (min/day)	561.0 (490.7–635.8)	571.9 (501.6–648.5)	549.0 (477.3–618.8)	543.9 (469–8-624.5)	554.7 (494.0–626.4)	561.7 (484.6–620.3)	564.1 (490.7–645.8)	590.3 (516.4–669.6)	564.0 (497.0–640.7)
Light physical activity (min/day)	306.1 (250.7–373.4)	301.8 (239.9–378.1)	322.3 (257.4–392.1)	316.4 (265.1–389.0)	295.4 (243.5–362.6)	286.1 (234.8–352.4)	309.6 (252.1–376.2)	307.7 (251.7–374.2)	300.2 (250.1–354.9)
Moraderate physical activity (min/day)	27.7 (16.2–45.6)	26.7 (15.6–44.4)	25.4 (15.4–43.1)	33.2 (22.6–50.5)	30.2 (16.1–44.3)	24.7 (12.8–41.8)	30.8 (17.9–52.8)	29.3 (16.5–51.7)	24.7 (14.3–42.3)
Vigorous physical activity (min/day)	0.0 (0.0–0.3)	0.0 (0.0–0.2)	0.0 (0.0–0.3)	0.0 (0.0–0.5)	0.0 (0.0–0.3)	0.0 (0.0–0.4)	0.0 (0.0–0.4)	0.0 (0.0–0.2)	0.0 (0.0–0.2)
MVPA (min/day)	28.3 (16.4–46.4)	27.6 (15.7–44.8)	26.3 (15.7–44.2)	33.5 (22.7–51.0)	30.6 (16.1–45.0)	25.7 (12.8–43.8)	31.3 (17.9–53.1)	29.7 (16.5–51.8)	24.7 (14.3–42.3)
Steps (counts/day)	9697.8 (6747.3–14,008.2)	7597.7 (5655.8–9651.8)	14,075.6 (10,146.4–17,243,6)	14,741.1 (12,115.5–17,726.0)	7507.7 (5741.8–9954.6)	7086.1 (5269.3–9091.8)	7659.0 (5824.3–102,264)	7954.8 (5978.1–10,510.3)	12,081.7 (8554.1–15,620.8)
Not meeting MVPA guidelines (%)	35.8	39.4	39.3	21.5	33.2	42.5	30.9	33.1	43.2

*SD* Standard deviation, *IQR* Interquartile range, *MVPA* Moderate-to-vigorous physical activity

Brazil, Colombia, Ecuador, Peru, and Venezuela were classified as high, and Argentina, Chile, and Costa Rica were classified as very high HDI. The HDI scores ranged from 0.759 for Ecuador to 0.985 for Chile. Significant differences between high and very high HDI countries were observed for age, ethnicity, vigorous physical activity and steps/day. No significant differences between HDI countries were observed for sedentary time, light, moderate, and moderate-to-vigorous physical activity (Table 2).

**Table 2:** Characteristics (% or median [IQR]) of the sample by human development index country

Variables	High human development index	Very high human development index	*p*-value
N	1.730	792	
Country (n)	5	3	
Sex (%)			0.274^b^
Men	48	45	
Women	52	55	
Age (years)	37.9	39.3	0.010^a^
Ethnicity (%)			0.002^b^
Mixed/caucasian	54	43	
Black	9	1	
White	29	49	
Other	8	7	
Accelerometer data			
Sedentary time (min/day)	560.9 (493.2–638.9)	561.1 (485.3–629.7)	0.221^a^
Light physical activity (min/day)	308.1 (251.8–374.4)	304.0 (248.2–374.3)	0.754^a^
Moderate physical activity (min/day)	27.3 (15.8–45.8)	28.7 (17.0–45.3)	0.390^a^
Vigorous physical activity (min/day)	0.0 (0.0–0.20)	0.0 (0.0–0.3)	0.040^a^
MVPA (min/day)	27.8 (15.9–46.6)	29.7 (17.1–46.0)	0.296^a^
Steps (counts/day)	9950.2 (6880.3–14,124.8)	9154.4 (6432.8–13,389.7)	0.019^a^

*MVPA* Moderate-to-vigorous physical activity

^a^ Mann–Whitney test (continuous variables)

^b^ chi square (categorical variables)

*p* < 0.05 for comparisons between high and very high human development index

Table 3 shows the results of the multivariate linear regression models for the effects of socio-demographic correlates and HDI on sedentary time and physical activity intensity, independent of country, region, and city. Women (-18.5 min/week) and participants of younger age (-0.7 min/week) presented lower sedentary time than men and those of older age. Participants of younger age (1.5 min/week), on the other hand, showed higher light physical activity than those of older age.

**Table 3:** Multivariate (β unstandardized coefficient) models for physical activity intensity

Predictors	Sedentary time (min/week)			Light physical activity (min/week)			Moderate physical activity (min/week)		
	Estimates	95%CI	*p*-value	Estimates	95%CI	*p*-value	Estimates	95%CI	*p*-value
Sex (ref. men)	-18.53	-28.16; -8,89	< 0.001	6.37	-0.71; 13.45	0.078	-12.62	-14.52; 10.73	< 0.001
Age (years)	-0.69	-1.05; -0.33	< 0.001	1.55	1.29; 1.82	< 0.001	-0.09	-0.17; -0.02	0.009
Ethnicity (ref. Mixed)									
Black	9.03	-11.35 – 29.42	0.385	7.29	-7.70 – 22.27	0.340	1.16	-2.86 – 5.18	0.571
Other	-3.70	-22.28 – 14.88	0.696	-3.36	-17.03 – 10.30	0.630	-1.01	-4.67 – 2.65	0.589
White/caucasian	0.49	-10.19 – 11.18	0.928	-4.28	-12.14 – 3.57	0.285	-2.76	-4.86 – -0.65	0.010
Human development index (country; ref. high)	-1.21	-12.03; 9.62	0.827	-4.25	-12.21; 3.71	0.295	1.31	-0.82; 3.45	0.227
	Vigorous physical activity (min/week)			Moderate-to-vigorous physical activity (min/week)			Steps (count/week)		
	Estimates	95%CI	*p*-value	Estimates	95%CI	*p*-value	Estimates	95%CI	*p*-value
Sex (ref. men)	-0.74	-0.94 – -0.54	< 0.001	-13.36	-15.32 – -11.41	< 0.001	-1266.48	-1667.83 – -865.14	< 0.001
Age (years)	-0.02	-0.03 – -0.01	< 0.001	-0.11	-0.19 – -0.04	0.002	12.89	-2.14 – 27.92	0.093
Ethnicity (ref. Mixed)									
Black	0.09	-0.34 – 0.51	0.683	1.25	-2.89 – 5.39	0.554	2853.90	2004.68 – 3703.13	< 0.001
Other	0.51	0.13 – 0.90	0.009	-0.50	-4.27 – 3.28	0.796	1785.45	1011.20 – 2559.70	< 0.001
White/Caucasian	0.06	-0.17 – 0.28	0.613	-2.70	-4.87 – -0.53	0.015	660.60	215.46 – 1105.75	0.004
Human development index (country; ref. high)	0.26	0.03 – 0.49	0.024	1.57	-0.62 – 3.77	0.161	-847.28	-1298.31 – -396.26	< 0.001

β regression coefficient with sedentary time and physical activity intensity (min/week) as dependent variable; adjustment: country, region, and city; *CI* Confidence interval, *Ref*: Reference, *p* < 0.05

Overall, women (-12.6 min/week), participants of younger age (-0.1 min/week), and those of white/caucasian ethnicity (-2.8 min/week) presented lower moderate physical activity than men, older age and mixed ethnicity. Women (-0.7 min/week) and participants of younger age (-0.1 min/week) also showed lower vigorous physical activity than men and older age. On the other hand, another ethnicity (0.5 min/week) and very high HDI (0.3 min/week) was associated with higher vigorous physical activity than mixed ethnicity and high HDI. For moderate-to-vigorous physical activity, we found lower levels in women (-13.4 min/week), participants of younger age (-0.1 min/week), and white/caucasian (-2.7 min/week) compared to men, those of older ages and with mixed ethnicity. Overall, women (-1266.5 steps/week) and participants from countries with very high HDI (-847.3 steps/week) presented lower steps than men and those from high HDI. On the other hand, black (2853.9 steps/week), other (1785.4 steps/week) and white/caucasian ethnicity (660.6 steps/week) were associated with higher steps compared to mixed ethnicity, independently of country, region, and city (Table 3).

## Discussion

This study aimed to analyse the relationship between socio-demographic correlates and HDI with sedentary time and physical activity intensity. Our analysis, including data from 2522 adults (18–65 years) from Latin America, showed lower moderate and vigorous physical activity in women, participants of younger age and those of white/caucasian ethnicity. Further, participants from very high HDI countries showed higher vigorous physical activity and lower steps/week, respectively.

The sex disparity regarding physical activity level has been explored in different studies. Women are more inactive among adolescents [13] and adults [30]. Among adults, the result are controversial [31]. In a worldwide epidemiological study, including just Brazil from Latin America, the difference of physical inactivity between genders was more evident among HDI countries, with women being more inactive than men. In contrast, in the high HDI countries, the prevalence of physical inactivity was greater among men [11]. Gender inequality seems to be the key to understand the difference between sex regarding physical activity. In addition, gender inequality, concerns about stereotypes due to of insecurities around body image are important barriers [32]. Interventions to improve women's physical activity are needed, particularly in countries with lower HDI. In countries with higher HDI, women might have more opportunities to be active because of their purchasing power, and in many cases, there are lower crime rates, which allows women to engage in leisure-time physical activity outside the home.

The relationship between HDI and physical inactivity was previously explored. It showed a higher prevalence of physical inactivity in low HDI countries, although the study relied on self-reported physical activity [11]. On the other hand, based on World Bank Income, the highest income countries present a higher prevalence of not meeting physical activity recommendations based on self-reported physical activity [33]. Additionally, a worldwide epidemiological study with 168 countries showed that the prevalence of physical inactivity was more than twice as high in high-income countries than in low-income countries [30] and the highest levels of physical inactivity were observed in Latin American and Caribbean women. Among adolescents, the prevalence of engaging in physical activity 5 to 6 days/week was higher in countries with the highest HDI [13]. The discrepancy between studies can be explained, in part, based on compositional differences in the study sample and physical activity measurement method. These different results highlight an area for future studies to understand better the factors affecting the relationship between physical activity and HDI. Our study exposed that HDI presents an association with vigorous physical activity and steps/week. But we did not find an association between HDI and sedentary time, moderate and moderate-to-vigorous physical activity. Vigorous physical activity is prevalent among sports activities, practices in health clubs, gymnasiums and other private places. It can be assumed that the opportunity to be engaged with vigorous physical activity is better among very high HDI countries. Also, very high HDI countries have better built environment that encourages walking. Many studies have reported a positive association between the built environment and physical activity [34–36].

Along with previous studies, the results of the present study have several practical implications for public health policies. There is a need for a stronger investment in programs looking for gender equity, in general and specifically in the physical activity field. It could also help guide better access to physical activity in countries considering social inequity. Potential interventions aiming to increase physical activity should also consider variations in socio-demographic correlates and HDI and focus on groups with lower moderate-to-vigorous physical activity levels including those of white/caucasian ethnicity. In addition, local differences between countries need to be considered. For instance, Argentina, Brazil, Costa Rica and Venezuela presented higher proportion of white/caucasian ethnicity participants.

The strengths of this study included the large sample size with participants from eight countries from Latin America. There are relatively few studies that have objectively assessed sedentary time and physical activity intensities in Latin America since most international epidemiological studies have employed self-report methods [5, 18]. Objective assessments for sedentary time and physical activity are rare for population health surveys. The best available evidence must be used to support and guide action to decrease sedentary time and increase physical activity levels. A limitation of our study included the cross-sectional design, which prevents conclusions regarding causality from being established. Our evaluation of accelerometer-measured daily activity may also not represent the total population in the eight participating countries since participants were recruited from specific urban neighborhoods. Furthermore, accelerometers do not capture common activities such as cycling, resistance and static exercise, and carrying loads [37].

## Conclusion

Different socio-demographic correlates are associated with physical activity intensity; There is also evidence for a country-specific influence of HDI on vigorous physical activity and steps per week in the Latin American region, which can guide policies to promote physical activity in the region. This process, initiated with national or regional physical activity surveillance, ultimately aims to improve physical activity levels and promote healthy lifestyles among Latin American adults.

## Data Availability

The datasets generated and/or analyzed during the current study are not publicly available due the terms of consent/assent to which the participants agreed but are available from the corresponding author on reasonable request. Please contact the corresponding author to discuss availability of data and materials.
